# Mitochondrial function in the heart: the insight into mechanisms and therapeutic potentials

**DOI:** 10.1111/bph.14431

**Published:** 2018-08-02

**Authors:** Binh Yen Nguyen, Andrea Ruiz‐Velasco, Thuy Bui, Lucy Collins, Xin Wang, Wei Liu

**Affiliations:** ^1^ Faculty of Biology, Medicine and Health The University of Manchester Manchester UK

## Abstract

Mitochondrial dysfunction is considered as a crucial contributory factor in cardiac pathology. This has highlighted the therapeutic potential of targeting mitochondria to prevent or treat cardiac disease. Mitochondrial dysfunction is associated with aberrant electron transport chain activity, reduced ATP production, an abnormal shift in metabolic substrates, ROS overproduction and impaired mitochondrial dynamics. This review will cover the mitochondrial functions and how they are altered in various disease conditions. Furthermore, the mechanisms that lead to mitochondrial defects and the protective mechanisms that prevent mitochondrial damage will be discussed. Finally, potential mitochondrial targets for novel therapeutic intervention will be explored. We will highlight the development of small molecules that target mitochondria from different perspectives and their current progress in clinical trials.

**Linked Articles:**

This article is part of a themed section on Mitochondrial Pharmacology: Featured Mechanisms and Approaches for Therapy Translation. To view the other articles in this section visit http://onlinelibrary.wiley.com/doi/10.1111/bph.v176.22/issuetoc

**Abbreviations**
∆Ψ: membrane potential
AAV: adeno‐associated virus serotypes
ANT: adenine nucleotide translocase
Apaf1: apoptotic protease activator‐1
ATF2: activating transcription factor 2
CsA: cyclosporine A
CPT1: carnitine palmitoyltransferase‐1
CREB: cAMP response element‐binding protein
CypD: cyclophilin D
Drp1: dynamin‐1‐like‐protein
EMRE: essential MCU regulatory element
ER: endoplasmic reticulum
ERR: oestrogen‐related receptors
ETC: electron transport chain
FAO: fatty acid β‐oxidation
Fis1: fission‐protein 1
GPX: glutathione peroxidase
I/R: ischaema reperfusion
IMM: inner mitochondrial membrane
LC3: light chain 3
Letm1: Leucine zipper and EF‐hand containing transmembrane protein 1
MCU: mitochondrial calcium uniporter
MCUR: MCU regulator
MEF2: myocyte enhancer factor 2
Mfn1/2: mitofusin 1/2
Mff: mitochondrial fission factor
MICU1/2: mitochondrial calcium uptake1/2
Mid49/51: mitochondrial elongation factor 1 and 2
MLKL: mixed lineage kinase domain‐like protein
MnSOD: mitochondrial antioxidant manganese SOD
mPTP: mitochondrial permeability transition pore
mtDNA: mitochondrial DNA
NCLX: Na^+^/Ca^2+^/Li^+^ exchanger
NR: nicotinamide riboside
NRF: nuclear respiratory factors
O₂^−^: superoxide
OMM: outer mitochondrial membrane
Opa1: optic atrophy 1
OSCP: oligomycin sensitivity‐conferring protein
PA: phosphatidic acid
PDHK: pyruvate dehydrogenase kinase
PE: phosphatidylethanolamine
PGC1α: PPARγ coactivator 1
PS: phosphatidylserine
PTM: posttranslational modification
RaM: rapid mode of Ca^2+^ uptake
RIPK1: receptor‐interacting kinase‐1
SIRT1/2/3: sirtuin 1/2/3
SLC25A23: solute carrier family 25 member 3
SMAC: second mitochondria‐derived activator of caspase
SNO: S‐Nitrosylation
TCA: tricarboxylic acid cycle
TFAM: mitochondrial transcription factor A
VDAC: voltage‐activated anion channel
XIAP: X‐linked inhibitor of apoptosis protein

## Introduction

The heart permanently consumes large quantities of energy, predominantly for contraction and ion transport purposes. However, its capacity to store energy is unexpectedly low. Hence, to maintain this high energy flux, http://www.guidetopharmacology.org/GRAC/LigandDisplayForward?ligandId=1713 must be constantly and rapidly synthesized. In cardiac myocytes, mitochondria occupy approximately one third of the cell volume reflecting the high energy demands of these cells. Mitochondria produce more than 95% of the ATP in the myocardium; in addition, mitochondria also play important roles in regulating redox status, calcium homeostasis and lipid synthesis. It is therefore not surprising that mitochondrial dysfunction has been strongly linked to the development of cardiomyopathy and an increased risk of heart failure (Murphy *et al*., 2016).

## Mitochondrial functions in the heart

### The powerhouse in cardiac cells

**Figure 1:**
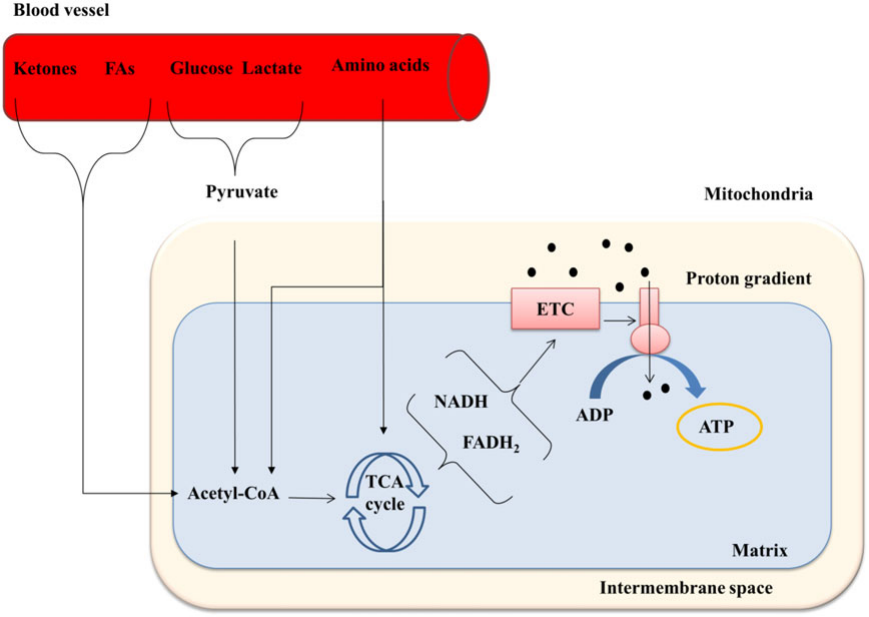
The main mitochondrial function is energy production. Mitochondria transform different substrates into ATP by driving the TCA cycle that supplies electron carriers. The electron carriers, NADH and FADH_2_ then stimulate the ETC to maintain a constant flux of protons towards the intermembrane space. The gradient generated by this flux is the force that powers ATP synthase. Mitochondrial substrates do not enter directly into the TCA cycle. Fatty acids and ketones are oxidized inside the mitochondria and broken into acetyl‐CoA. Glucose and lactate are converted into pyruvate in the cytosol, and pyruvate is converted into acetyl‐CoA in the mitochondrial matrix. Amino acids can supply not only acetyl‐CoA but also other TCA intermediates.

To provide a constant supply of energy, and at the same time adapt to stimuli, mitochondria transform different substrates available into ATP. This substrate plasticity accommodates changes in the environment, such as oxygen and nutrient availability in blood, and rapid changes in workload, such as during exercise. Fatty acids are the preferred substrate, accounting for 60–90% of the myocardium's energy supply (Murphy *et al*., 2016). Once fatty acids are taken from the bloodstream, they are transported into the mitochondria for fatty acid β‐oxidation (FAO). In this set of reactions, the long‐chain fatty acids are broken down into acetyl‐CoA, which then enters the tricarboxylic acid cycle (TCA), also known as Kreb's cycle. Electron carriers, FADH_2_ and http://www.guidetopharmacology.org/GRAC/LigandDisplayForward?ligandId=4487, produced during the TCA cycle transfer electrons into the electron transport chain (ETC). This, in turn, drives protons into the intermembrane space to activate the ATP synthase while consuming oxygen. In contrast, glucose is first transformed into http://www.guidetopharmacology.org/GRAC/LigandDisplayForward?ligandId=4809 in the cytosol, a process denominated glycolysis. The mitochondrial complex pyruvate dehydrogenase converts pyruvate into acetyl‐CoA, in turn feeding the TCA cycle and ETC (Figure 1). Fatty acid and glucose metabolisms regulate one another in a negative feedback cycle, known as the Randle cycle, which ensures optimal use of the resources available at all times (Hue and Taegtmeyer, 2009). Other energy substrates, such as ketones, amino acids and lactate, can also be oxidized to sustain the TCA cycle and produce ATP (Murphy *et al*., 2016). Their contribution to overall myocardial energetics at rest is minor but becomes important under different stress conditions.

### Regulation of redox status

Cardiovascular diseases are closely associated with oxidative stress triggered by elevated levels of ROS. The accumulation of ROS in cells is a primary cause of mitochondrial damage and dysfunction. Indeed, mitochondria are the main cellular source of ROS. Electrons from coenzymes (NADH and FADH_2_) are tightly coupled to oxidative phosphorylation for ATP synthesis and are driven to oxygen by the ETC to release water. Nevertheless, about 0.2–2% of electrons leak from the ETC and are aberrantly transferred to O_2_, resulting in the formation of superoxide (O_2_^−^). Electron leakage happens at all three complexes I, II and III. Superoxide releases from complexes I and II into the matrix and from complex III to the intermembrane space and the matrix (Murphy *et al*., 2016).

In mitochondria, ROS production is counterbalanced by efficacious detoxification defence. In the matrix compartment, superoxide can be further transformed into http://www.guidetopharmacology.org/GRAC/LigandDisplayForward?ligandId=2448 by the mitochondrial antioxidant manganese SOD (MnSOD, SOD2) (Bhattacharyya *et al*., 2014). MnSOD‐deficient mice die 10 days postnatally and exhibit cardiomyopathy. Inhibition of MnSOD activities in isolated cardiomyocytes also increases the levels of apoptosis and hypertrophy (Gustafsson and Gottlieb, 2008). Moreover, H_2_O_2_ can either cross mitochondrial membranes freely or can be further detoxified to water by other mitochondrial antioxidant enzymes, such as glutathione peroxidase (GPX) in the presence of http://www.guidetopharmacology.org/GRAC/LigandDisplayForward?ligandId=6737 (Bhattacharyya *et al*., 2014).

At low levels, ROS can serve as a signalling molecule to modify signalling proteins and have functional consequences (Murphy *et al*., 2016). On the contrary, during cardiovascular diseases, overproduction of ROS is accompanied by reduced antioxidant capacity and oxidative stress. For example, MnSOD and GPX activities have been shown to be reduced significantly in animal models of congestive heart failure and myocardial infarction (Gustafsson and Gottlieb, 2008). Excessive ROS results in the shutdown of energy production, increased cell death and irreversible oxidative damage to mitochondrial DNA (mtDNA) and altered gene expression. Consequently, this results in the development and progression of cardiac dysfunction.

### Homeostasis of calcium

Mitochondria are also responsible for regulating the transport of ions, and their numerous ion channels and exchangers may be important for protecting cells. The most important ones are the selective K^+^ and Ca^2+^ channels, the Na^+^/H^+^ exchanger and the K^+^/H^+^ exchanger. The correct function of these ion channels is required to preserve mitochondrial membrane potential, which supports the production of ATP and redox regulation (Murphy *et al*., 2016).

Among these ions, Ca^2+^ plays a central role in the regulation of many cellular functions including metabolism and the effects of transcription‐modifying enzymes (Stefani *et al*., 2016). In cardiomyocytes, excitation‐contraction coupling during contraction is accompanied by a surge in cytosolic calcium, which leads to ensuing calcium removal from cytosol during subsequent relaxation. As they contain several different ion channels and transporters, mitochondria are capable of sensing cytosolic calcium signals and mediating calcium sequestrion from the cytosol into the mitochondrial matrix. Ca^2+^ is able to easily diffuse through the outer mitochondrial membrane (OMM) *via* voltage‐dependent anion channels (VDACs) (Arruda and Hotamisligil, 2015). In contrast, the inner mitochondrial membrane (IMM) is impermeable to ions, emphasizing the need to identify mitochondrial Ca^2+^ transporter proteins. The identification of the mitochondrial calcium uniporter (MCU) on IMM as a central mediator of mitochondrial calcium influx has made the field more attractive and challenging (Baughman *et al*., 2011; Stefani *et al*., 2011). MCU is a 40 kDa protein comprised of two coiled‐coil domains and two transmembrane helices separated by a 9‐amino acid loop and is characterized by its low affinity for binding Ca^2+^. Despite that, siRNA‐mediated knockdown of MCU led to an attenuated mitochondrial calcium uptake (Baughman *et al*., 2011; Stefani *et al*., 2011). Isolated mitochondria from MCU‐deleted mice demonstrated attenuated calcium uptake and a decreased level of calcium in the mitochondrial matrix (Pan *et al*., 2013). Further studies revealed the other two core‐components of the MCU complex: MCUb and essential MCU regulatory element (EMRE). MCUb, a 33 kDa protein, shares 50% homology with MCU and is capable of disrupting MCU‐mediated Ca^2+^ permeability; whilst EMRE is a 10 kDa protein and absolutely pivotal for the assembly of the MCU complex (Stefani *et al*., 2016).

In addition to the structural components, MCU activities are tightly controlled by two regulatory proteins: mitochondrial calcium uptake 1 (MICU1) and mitochondrial calcium uptake 2 (MICU2). These proteins form a heterodimer that is linked with MCU *via* EMRE. Several studies have implied that MICU1 and MICU2 function as Ca^2+^ sensors by closing MCU pores when cytosolic [Ca^2+^] is low. MICU1‐ablation in mitochondria led to matrix Ca^2+^ overload and less efficient Ca^2+^ uptake (Liu *et al*., 2016; Stefani *et al*., 2016). Therefore, as a MCU gatekeeper, MICU1/2 prevent excessive accumulation of Ca^2+^ within the mitochondrial matrix, preserving proton motive force. Additionally, MCU regulator 1 (MCUR1) and the mitochondrial nucleotide transporter http://www.guidetopharmacology.org/GRAC/FamilyDisplayForward?familyId=206#1078 have also recently been reported to regulate calcium homeostasis. Although deletion of both MCUR1 and SLC25A23 results in reduced MCU‐mediated Ca^2+^ intake, the precise mechanisms underlying the regulation of MCU activities remains unknown (Stefani *et al*., 2016).

MCU‐independent and rapid mechanisms have been studied. In contrast to MCU, these routes mediate faster Ca^2+^ uptake than MCU due to a high affinity for Ca^2+^ and are inhibited by high Ca^2+^ concentrations (Ryu *et al*., 2010). Among these is the rapid mode of Ca^2+^ uptake (RaM), which was initially recognized in isolated heart and liver mitochondria. Although the kinetic mode of RaM has been demonstrated to occur only transiently during the beginning of calcium pulses, its molecular identity has remained unclear (Ryu *et al*., 2010). Moreover, mitochondrial ryanodine receptors are IMM proteins that are widely accepted as an alternative for rapid Ca^2+^ import (Ryu *et al*., 2010). Recently, a mitochondrial protein leucine zipper and EF‐hand containing transmembrane protein 1 (Letm1), originally known as the K^+^/H^+^ exchanger, has been demonstrated as an important mitochondrial Ca^2+^/H^+^ antiporter, which extrudes H^+^ from the matrix in exchange for Ca^2+^. One study has demonstrated that *in vitro* deletion of *Letm1* leads to impaired Ca^2+^ uptake and reduced ATP production, whereas *in vivo* ablation of *Letm1* is lethal (Jiang *et al*., 2013).

Mitochondrial Ca^2+^ influx is counteracted by the IMM http://www.guidetopharmacology.org/GRAC/FamilyDisplayForward?familyId=202#1050, which is able to extrude Ca^2+^ in exchange for Na^+^ and Li^+^. Mice with cardiac‐specific deletion of NCLX have increased lethality due to severe cardiac remodelling, which are attributed to mitochondrial Ca^2+^ overload along with augmented ROS production (Luongo *et al*., 2017). Interestingly, NCLX activity has been reported to serve in a reverse manner to take Ca^2+^ into the mitochondrial matrix; therefore, more studies are required to unravel this concept (Stefani *et al*., 2016).

### Lipid synthesis

Mitochondria also have a crucial role in lipid homeostasis. Most lipids are synthesized in the endoplasmic reticulum (ER); however, some major components of the mitochondrial membranes are synthesized in IMM from imported lipids. http://www.guidetopharmacology.org/GRAC/LigandDisplayForward?ligandId=3638) and phosphatidic acid (PA) are imported from the ER membrane to the OMM through membrane contact sites called mitochondrial‐associated ER membranes. The lipid precursors are then transferred across the intermembrane space by protein complexes, such as the ubiquitin specific peptidase 1/2‐mitochondrial distribution and morphology protein 35 complex, or the PRELID1‐TRIAP1 complex in humans (Mesmin, 2016). When reaching the IMM, phosphatidylserine decarboxylase transforms PS to phosphatidylethanolanime (PE); meanwhile, PA goes through a series of reactions and is converted to the phospholipid cardiolipin. Both phospholipids are especially abundant in IMM and are essential for cristae structure. PE plays a role in membrane protein folding (Bogdanov and Dowhan, 1999) but, more importantly, has been observed to be necessary for efficient mitochondrial respiration (Tasseva *et al*., 2013). Moreover, cardiolipin has been found to be distributed towards the OMM through mitochondrial contact sites that hold the IMM and OMM close together and maintain structural stability. The transport of cardiolipin to the OMM has been observed to be increased in response to stress, serving as a binding site for signalling molecules in mitophagy (Chu *et al*., 2013).

## Mitochondrial alterations in cardiac diseases

### Substrate shifts

**Figure 2:**
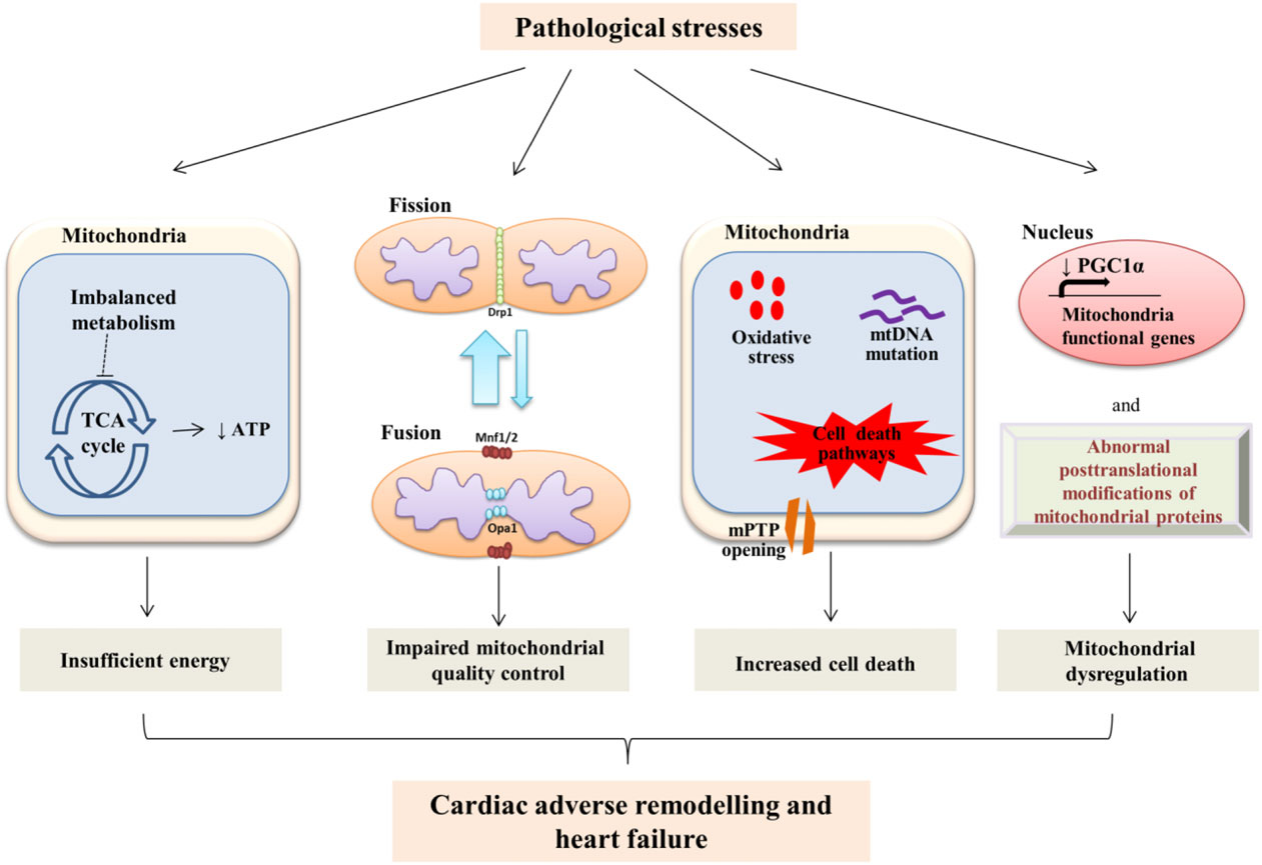
Mitochondrial dysregulation under pathological stresses. Sustained pathological stresses result in alterations in mitochondrial structure and function through different mechanisms, eventually leading to cardiac pathological remodelling and heart failure. Firstly, aberrant metabolic substrate preference in the heart impairs oxidative phosphorylation and ATP production, resulting in energy insufficiency. Secondly, an imbalance between mitochondrial fission and fusion induces the accumulation of fragmented mitochondria and impaired mitochondrial quality control, which ultimately disrupts the mitochondrial network and functions. Thirdly, excessive ROS production, increased mtDNA damages, activated cell death pathways and mPTP opening are the main causes of cell death. Finally, a dysregulation of transcription factors and abnormal posttranslational modification of mitochondrial function‐related genes impairs mitochondrial biogenesis.

Although fatty acids are the main substrate for ATP production in the myocardium, it must rely on different substrates to respond to and compensate for stress conditions. For example, during strenuous exercise, lactate consumption has been observed to significantly increase to cover the demand for rapid and higher energy production (Brown *et al*., 2017). In pathology, the substrate balance is initially shifted towards a temporarily more efficient profile, but later, this shift results in a deficiency in ATP that contributes to heart failure (Figure 2). In high‐fat‐diet‐induced hypertrophy and diabetes, an increase in FAO is observed during early stages, whereas pressure overload and ischaemia favour glucose metabolism (Kolwicz *et al*., 2013). The increase in either situation strongly inhibits the utilization of the other substrates by the Randle cycle, thereby reducing the myocardium's ability to process different substrates. For example, non‐diabetic hypertrophy models relying on glucose oxidation develop insulin resistance, which limits substrate plasticity (Nikolaidis *et al*., 2004). In contrast, diabetic models with a high FAO rate accumulate lipid metabolites that generate mitochondrial dysfunction, a process called lipotoxicity (Bayeva *et al*., 2013b). In advanced stages of the disease, both fatty acid and glucose metabolisms are reduced. The myocardium turns to ketone utilization for ATP production but still faces energy deprivation (Brown *et al*., 2017). The myocardium requires the ability to process different substrates in order to maintain proper function.

### Alterations in mitochondrial dynamics

The balance between mitochondrial fission and fusion maintains its normal dynamics under physiological conditions, which contributes largely to the regulation of cell survival and cellular metabolism. In addition, well‐controlled mitochondrial dynamics are critical for the maintenance of mitochondrial morphology and distribution, which is strictly regulated based on cellular demands (Murphy *et al*., 2016). Mitochondrial fission is activated during cell division to generate healthy daughter mitochondria through replicative fission for higher energy demand. Mitochondrial fission is initiated by the recruitment and oligomerization of the GTPase from the dynamin family, dynamin‐1‐like‐protein (Drp1), onto the OMM to form helical rings encircling mitochondria. Upon the hydrolysis of http://www.guidetopharmacology.org/GRAC/LigandDisplayForward?ligandId=1742, the ring‐like structure constricts to split the OMM and IMM (Ong *et al*., 2015). In addition, several mitochondrial outer membrane proteins including mitochondrial fission protein‐1 (Fis1), mitochondria fission factor (Mff) and mitochondrial elongation factor 1 and 2 (MiD49 and MiD51) are required to mediate the assembly of the mitochondrial fission machinery. Mff and MiD49/51 are proposed as the *bona fide* Drp1 receptors to recruit Drp1 onto the OMM, whereas the specific roles of Fis1 are still controversial. Although early studies, using Fis1 overexpression and inhibition, support pivotal roles of Fis1 during mitochondrial fission, a follow‐up investigation in cancer cell lines shows that *Fis1* deletion does not impair Drp1 recruitment and mitochondrial morphology (reviewed in detail by Chan, 2012). However, mitochondrial fusion is enhanced in response to pathological stress to prevent damage to mtDNA and mitochondrial proteins. This process is regulated by members of the dynamin protein family, namely, mitofusin 1/2 (Mfn1/2) and optic atrophy 1 (Opa1), which work in collaboration to mediate fusion of both OMM and IMM (Ong *et al*., 2015).

Rat and human failing hearts exhibit mitochondrial fragmentation and decreased levels of fusion proteins while fission protein levels are increased (Chen *et al*., 2009; Javadov *et al*., 2011). These data might indicate the predominance of fission over fusion during heart failure by which the heart function is worsened (Figure 2). Alterations in mitochondrial dynamic proteins trigger excessive ROS generation, abnormal enzymatic activities and impaired calcium homeostasis, eventually leading to cardiac dysfunction. For example, Drp1 ablation results in elongated mitochondria and dilated cardiomyopathy; whereas mice with deletion of Mfn1/2 develop eccentric hypertrophy along with the accumulation of fragmented mitochondria (Song *et al*., 2015b; Song *et al*., 2017). Additionally, hearts from heterozygous Opa1^+/−^ mice display decreased fractional shortening and reduced cardiac output associated with impaired mitochondrial function (Chen *et al*., 2012). Consistently, knockdown of Opa1 or Drp1 reduces oxygen consumption and the rate of ATP synthesis (Chen *et al*., 2005; Benard *et al*., 2007). Further study has identified that stress‐induced metalloendopeptidase OMA1 deactivates Opa1 activity, which leads to mitochondrial fragmentation, cardiac metabolic shift towards glucose utilization and dilated cardiomyopathy (Wai *et al*., 2015). Therefore, a balance between mitochondrial fission and fusion proteins is required to maintain cardiac function.

### Impairments in the quality control of mitochondria

Mitochondria have developed mechanisms to control and maintain their quality at appropriate levels through the balance of biogenesis and mitophagy. In contrast to mitochondrial biogenesis, which facilitates repopulation with new healthy mitochondria through fission–fusion cycle, mitophagy is a cellular process that specifically identifies and eliminates damaged and dysfunctional mitochondria. Mitophagy is mediated by the cytosolic E3 ubiquitin ligase Parkin and the mitochondrial kinase http://www.guidetopharmacology.org/GRAC/ObjectDisplayForward?objectId=2161. Activation of the PINK1–Parkin pathway is tightly controlled and regulated by the mitochondrial membrane potential (∆Ψ), which is a driving force for ATP synthesis (Murphy *et al*., 2016). In healthy and hyperpolarized mitochondria, PINK1 activity is maintained at a low level, whereas damaged mitochondria with abnormal ∆Ψ display inhibited PINK1 degradation, leading to its accumulation and stabilization at OMM. In addition, Wang *et al*., 2018 demonstrated that http://www.guidetopharmacology.org/GRAC/ObjectDisplayForward?objectId=1542 isoforms are required to phosphorylate PINK1 at Ser^495^, facilitating its stabilization and induction of mitophagy in response to transverse aortic constriction‐induced chronic heart failure. PINK1 recruits Parkin from the cytosol, allowing it to ubiquitylate mitochondrial proteins. As a result, Parkin‐mediated polyubiquitinated proteins function as receptors for protein‐1 light chain 3 (LC3) on the autophagosome, hence promoting autophagosomal engulfment of the depolarized mitochondria and commencing mitophagy (Shirihai *et al*., 2015). This pathway contributes largely to the mitochondrial quality control mechanism. Recently, a communicating mechanism between PINK1 and Parkin has been elucidated, which provides a link between Mfn2 and mitophagy. A deficiency in Mnf2 induces the accumulation of Parkin in cytosol, which results in reduced levels of mitophagy (Song *et al*., 2015b). Parkin‐mediated mitophagy is also closely associated with mitochondrial fission. Parkin is up‐regulated in Drp1‐ablated hearts, which leads to hyper‐mitophagy and dilated cardiomyopathy, while concomitant ablation of Parkin attenuated the pathological phenotypes caused by a deficiency in Drp1 (Song *et al*., 2015a).

Besides the conventional PINK1–Parkin pathway, PINK1‐independent mitophagy is considered to be a generalized mitochondrial autophagy. Several OMM proteins such as http://www.guidetopharmacology.org/GRAC/ObjectDisplayForward?objectId=2844&familyId=910&familyType=OTHER#OtherNames/adenovirus E1B 19 kDa protein‐interacting protein 3‐like/mitochondrial receptor Nip3‐like protein X (BNIP3L/NIX), Bcl‐2/adenovirus E1B 19 kDa protein‐interacting protein 3 (BNIP3) and FUN14 domain containing 1 (FUNDC1) have been shown to function as receptors for LC3 to trigger mitochondrial autophagy (Shirihai *et al*., 2015). Recently, Bhujabal *et al*. (2017) expanded the repertoire of PINK1‐independent mitochondrial autophagy by an anti‐apoptotic protein, FKBP8, which is able to interact with LC3 to induce autophagosomal engulfment. In addition, McWilliams *et al*., 2018 have pointed out that mitophagy happens preferentially through PINK1‐independent mechanisms at physiological conditions in high‐energy‐demanding tissues.

To date, several reports have focused on the protective roles of mitophagy in the heart in response to stress. For instance, hearts subjected to *ex vivo* ischaemia/reperfusion (I/R) injury showed enhanced mitophagy (Moyzis *et al*., 2015). PINK1‐deficient hearts are more susceptible to *ex vivo* I/R injury and develop hypertrophy in response to pressure overload more rapidly (Billia *et al*., 2011; Lee *et al*., 2011). Furthermore, the infarct size after I/R injury was exacerbated due to loss of PINK1 (Siddall *et al*., 2013). Additionally, Parkin‐deficient mice accumulate dysfunctional mitochondria and exhibit increased mortality following a myocardial infarction (Kubli *et al*., 2013). Interestingly, the translocation of Parkin to mitochondria was observed in ischaemic preconditioning (IPC), which was abolished in Parkin‐knockout mice (Huang *et al*., 2011). Although mitophagy has been studied for several decades, further studies are required to investigate whether mitophagy will be a promising target for cardioprotection.

### mtDNA mutations

mtDNA remains a key element of mitochondrial function. It encodes 37 essential mitochondrial proteins (POLG), including tRNAs, rRNAs and subunits of the enzyme complexes in *ETC*. mtDNA replication is performed by DNA polymerase γ, and its spontaneous mutation rate has been estimated to be much higher than replication of the nuclear genome. The frequency of a spontaneous pathogenic mtDNA mutation is estimated to be as high as 1 in 5000 individuals; therefore, it is not surprising that even if most of the severe mutations are selectively eliminated from the maternal germline in a few generations, some others can be conserved and result in cardiomyopathy (Fan *et al*., 2008). Moreover, the crosstalk between mitochondrial and nuclear genomes is necessary for normal mitochondrial performance and, ultimately, for optimal cardiac function. For example, a particular mutation in the nuclear gene, http://www.guidetopharmacology.org/GRAC/FamilyDisplayForward?familyId=206#1062, leads to hypertrophy or critical dilated cardiomyopathy depending on the patient's particular haplogroup (Strauss *et al*., 2013). In mice with ANT mutation, cardiomyopathy was associated with age‐related accumulation of somatic mtDNA mutations (Narula *et al*., 2011). Therefore, the interaction of mtDNA mutation with nuclear DNA mutation can exacerbate cardiac pathology.

## Regulation of mitochondrial biogenesis and metabolism

### Mitochondrial transcriptional network

#### Transcriptional regulation of mitochondrial genes

In response to greater energy expenditure, mitochondrial biogenesis is triggered within cells, leading to enrichment in their mitochondrial mass and number to increase ATP production. The process is a critical facilitator for mitochondrial homeostasis and metabolic enzyme activities, and it is intricately regulated at the transcriptional level by genes encoding mitochondrial proteins. A widely recognized master regulator of mitochondrial gene transcription in the nucleus is PPARγ coactivator 1 (PGC1α), which has been continuously identified as a key element in the progression of cardiomyopathy to heart failure (Rowe *et al*., 2010). Despite not having independent transcriptional activity, PGC1α regulates a transcriptional network integrated by nuclear respiratory factors (NRFs), http://www.guidetopharmacology.org/GRAC/ObjectDisplayForward?objectId=622s), and http://www.guidetopharmacology.org/GRAC/FamilyDisplayForward?familyId=86 to control substrate utilization, mitochondrial biogenesis and dynamics.

Cardiac function is dependent on this transcriptional network properly regulating mitochondrial function. Cardiac‐specific E3 ligase mitsugumin 53 overexpression in mice, which up‐regulates http://www.guidetopharmacology.org/GRAC/ObjectDisplayForward?objectId=593 expression, strongly promotes FAO and results in diabetic cardiomyopathy (Liu *et al*., 2015). In hypertrophied mouse hearts induced by angiotensin II infusion, reduced mitochondrial expression of PGC1α, ERR and mitochondrial transcription factor A (TFAM) correlated with mitochondrial damage and increased mitophagy (Dai *et al*., 2011). Of note, transcriptional factors mediating mitochondrial genes are required for fine‐tuned regulation. For example, different mouse models with global or cardiac‐specific deletion of PGC1α, PPAR and ERR have been generated, resulting in the development of cardiomyopathy, although murine models with constitutive overexpression of PGC1 and PPAR are associated with increased mitochondrial biogenesis, which also leads to cardiomyopathy and heart failure (Rowe *et al*., 2010). Additionally, NRFs bind to the promoters of ETC genes and mitochondrial transcription factor family genes (TFAM and TFB), which are in charge of mtDNA transcription and translation; therefore, achieving necessary crosstalk between nuclear and mitochondrial genomes. NRF (Ristevski *et al*., 2004) and TFAM (Larsson *et al*., 1998) deletions were embryonically lethal and displayed mtDNA depletion. However, TFAM overexpression did preserve mitochondrial content and function after myocardial infarction, improving cardiac function (Ikeuchi *et al*., 2005).

#### Transcriptional and posttranslational regulation of PGC1α

PGC1α expression and activity are constantly modified by extracellular stimuli and intracellular conditions. First of all, its expression is mostly regulated by transcriptional factors. In muscles, myocyte enhancer factor 2 (MEF2), cAMP response element‐binding protein (CREB) and activating transcription factor 2 (ATF2) increase PGC1α expression by binding to its promoter. They are independently activated or repressed by different signalling cascades. For example, during exercise, enhanced intracellular calcium initiates calcineurin A and Ca^2+^/calmodulin‐dependent protein kinase pathways, which subsequently activate MEF2 and CREB (Handschin *et al*., 2003). The MAPK http://www.guidetopharmacology.org/GRAC/ObjectDisplayForward?objectId=1499 phosphorylates MEF2 and ATF2 to stimulate mitochondrial biogenesis in response to exercise (Akimoto *et al*., 2005). Under high‐fat‐diet stress, the http://www.guidetopharmacology.org/GRAC/ObjectDisplayForward?objectId=2093‐MEF2 pathway is active to maintain PGC1α expression for preserving mitochondrial integrity (Liu *et al*., 2017). Of note, a depressed PGC1α transcriptional network is a feature of heart failure induced by pressure overload (Garnier *et al*., 2003) or myocardial infarction, and it correlates with a reduced mitochondrial respiratory capacity, even when mitochondrial mass is not decreased, which is observed in human hypertrophic myocardium and ischaemic‐induced failing heart (Pisano *et al*., 2016) (Figure 2).

Secondly, PGC1α is strongly regulated at the posttranslational level as well. Its activity is affected by phosphorylation, acetylation, methylation and ubiquitination. The ‘energy sensor', http://www.guidetopharmacology.org/GRAC/ObjectDisplayForward?objectId=1540, is activated by high ADP/ATP levels to phosphorylate PGC1α (Jäger *et al*., 2007). It is not clear how, but the ensuing increase in transcriptional activity and expression of PGC1α induced by AMPK is possibly through an auto‐regulatory effect (Handschin *et al*., 2003). This positive feedback amplifies the control of AMPK over PGC1α. In fact, AMPK stimulation preserves cardiac contractility after myocardial infarction by promoting PGC1α expression and augmenting mitochondrial respiration (Gundewar *et al*., 2009). PGC1α phosphorylation is also regulated by p38 (Adams *et al*., 1998). Acetylation of PGC1α is predominantly mediated by amino acid synthesis 5; whereas its increased activity *via* deacetylation is regulated by http://www.guidetopharmacology.org/GRAC/ObjectDisplayForward?objectId=2707). A down‐regulation of SIRT1 was observed in human failing or ageing hearts (Lu *et al*., 2014). Additionally, preconditioning before I/R injury increased SIRT1 expression associated with an up‐regulated antioxidant capacity and diminished apoptosis (Hsu *et al*., 2010). The roles of methylation and ubiquitination of PGC1α in the heart are still under study.

### Posttranslational modifications (PTMs) of mitochondrial proteins

The correct crosstalk between the nucleus, cytosol and mitochondria is carried out by PTMs of mitochondrial proteins by acetylation, phosphorylation and nitrosylation. First, acetylation of mitochondrial enzymes modulates FAO, ATP production, antioxidant response and apoptosis in response to nutrient availability and cardiac stress signals (Murphy *et al*., 2016). In addition to SIRT1 found in the nucleus, http://www.guidetopharmacology.org/GRAC/ObjectDisplayForward?objectId=2708 and http://www.guidetopharmacology.org/GRAC/FamilyDisplayForward?familyId=848#2709 are NAD+‐dependent deacetylases predominantly located in the cytosol and mitochondria respectively. Cardiomyocyte‐specific SIRT3 expression has been shown to protect the heart against pathological hypertrophy through activation of FoxO3‐dependent antioxidant genes (Sundaresan *et al*., 2009). Meanwhile, its deficiency resulted in increased vulnerability to I/R due to inhibition of MnSOD (Porter *et al*., 2014) and opening of the mitochondrial permeability transition pore (mPTP) (Parodi‐Rullán *et al*., 2017).

Secondly, phosphorylation of mitochondrial proteins plays an essential role in mitochondrial function, such as protein transporting, enzyme activation and substrate shift. AMPK phosphorylates a wide selection of enzymes to promote ATP synthesis. For example, it phosphorylates http://www.guidetopharmacology.org/GRAC/FamilyDisplayForward?familyId=255#1264 which stimulates FAO; moreover, it also enhances glucose metabolism by promoting http://www.guidetopharmacology.org/GRAC/FamilyDisplayForward?familyId=165#878 translocation and phosphorylating phosphofructokinase 2. With its diversity of targets, it is not surprising that AMPK manipulation has produced conflicting results; nevertheless, it is generally agreed that mild AMPK activation has cardioprotective effects and its deletion impairs energy metabolism (Zaha and Young, 2012). The http://www.guidetopharmacology.org/GRAC/FamilyDisplayForward?familyId=608), branched chain α‐ketoacid dehydrogenase kinase (http://www.guidetopharmacology.org/GRAC/ObjectDisplayForward?objectId=1939), and phosphatase are also known to control substrate shifts by phosphorylation of mitochondrial proteins. However, more than 150 proteins were identified to possess phosphorylation sites in cardiac mitochondria (Deng *et al*., 2011) suggesting future research will uncover further regulatory systems mediated by phosphorylation.

Thirdly, S‐nitrosylation (SNO) is the covalent attachment of NO to a cysteine residue. The mechanisms controlling this process are yet to be clarified; nevertheless, it has been observed that mitochondrial proteins are prone to be nitrosylated for their function. More importantly, SNO has been intimately linked to I/R injury and heart failure. Protein SNO was found to be decreased after I/R (Lima *et al*., 2010) and, in contrast, was increased during IPC. Augmented SNO resulting in improved cardiac function is associated with a preserved complex I respiration rate and ATPase function (Sun *et al*., 2007; Prime *et al*., 2009).

## Mechanisms of mitochondria‐mediated cell death in cardiac diseases

Cell death is a hallmark characteristic of heart diseases, and mitochondria contribute largely to cardiomyocyte death in response to pathological stresses. Distinct types of cell deaths including apoptosis, necrosis and necroptosis have been recognized during the progression of cardiac diseases (Figure 2).

### Apoptosis

Mitochondria are a well‐known regulator of intrinsic cardiomyocyte apoptosis. In response to death signals and ROS accumulation, mitochondria facilitate the release of apoptogens, such as cytochrome c, Omi/HtrA2, second mitochondria‐derived activator of caspase (SMAC or DIABLO) and apoptosis‐inducing factor (AIF) into cytosol from the mitochondrial intermembrane space (Murphy *et al*., 2016). Once in the cytosol, these apoptogens bind to apoptotic protease activator‐1 (Apaf1) to mediate assembly of the apoptosome for procaspase‐9 recruitment and activation (McIlwain *et al*., 2015). The initiator http://www.guidetopharmacology.org/GRAC/ObjectDisplayForward?objectId=1625 then cleaves inactive procaspase dimers to form active executioner http://www.guidetopharmacology.org/GRAC/ObjectDisplayForward?objectId=1619 and http://www.guidetopharmacology.org/GRAC/ObjectDisplayForward?objectId=1623. Upon activation, these executioner caspases can activate other caspases to induce a magnitude of caspase activation within the cell. Finally, these activated endoproteases take part in degrading structural proteins and enzymes to bring about cellular apoptotic responses, such as DNA fragmentation, membrane shrinkage and the formation of apoptotic bodies, for subsequent phagocytosis (Murphy *et al*., 2016).

The integrity of OMM is regulated by both anti‐apoptotic (Bcl‐2, Bcl‐X_L_) and pro‐apoptotic members (Bax, Bak) of the Bcl‐2 protein family. In response to apoptotic signals, Bax and Bak bind to BH‐3 proteins, such as Bim or truncated Bid, to assist their conformational changes. Once activated, cytosolic Bax translocates to OMM where it undergoes both homo‐oligomerization and hetero‐oligomerization with Bak. Meanwhile, anti‐apoptotic proteins, such as Bcl‐2 and Bcl‐X_L_, prevent Bax and Bad activation through direct interactions with Bim and truncated Bid in the cytosol (Murphy *et al*., 2016). Overall, the ratio between apoptotic and pro‐apoptotic protein levels within a cell determines OMM permeability and cytochrome c release to the cytosol.

Unlike other cell types, cardiomyocytes are highly resistant to caspase‐dependent apoptosis, which suggests the importance of caspase‐independent apoptotic mechanisms. This is due to a high expression level of the endogenous http://www.guidetopharmacology.org/GRAC/ObjectDisplayForward?objectId=2790) in cardiomyocytes, which prevents Apaf1 activity and caspase activation even in the presence of apoptotic stimuli (Potts *et al*., 2005). Moreover, under severe oxidative stress, mitochondrial serine protease HtrA2/Omi translocate from mitochondria to cytosol, where they promote apoptosis through protease‐dependent degradation of XIAP and caspase activation (Liu, 2005). By contrast, http://www.guidetopharmacology.org/GRAC/FamilyIntroductionForward?familyId=889 (IAPs), expressed in the heart as caspase 9 inhibitors, limit I/R‐induced apoptosis in isolated perfused mouse hearts (Chua *et al*., 2007).

Alterations in mitochondrial morphology also contribute to apoptotic responses in the heart. For example, angiotensin II‐induced hypertensive hearts demonstrate a downregulation of SIRT1, thus allowing p53 acetylation and Drp1‐dependent mitochondrial fission, from which mitochondrial fragmentation eventually results in cardiomyocyte apoptosis (Qi *et al*., 2018). Additionally, a down‐regulation of cardiac dual‐specificity protein phosphatase 1 in acute I/R injury activates mitochondrial fission and cardiomyocyte apoptosis (Jin *et al*., 2018). I/R injury can also bring about cardiac microcirculation collapse by facilitating Mff expression, which promotes the apoptosis of endothelial cells in the cardiac microcirculation through increased mPTP opening and cytochrome c release to the cytosol (Zhou *et al*., 2017). Taken together, morphological alterations in mitochondria are critical causes for stimulating apoptosis in cardiac diseases.

### Necrosis

In contrast to apoptosis, necrosis is a passive and unregulated form of cell death that is distinguished by distinctive mitochondrial alterations, such as mitochondrial swelling, loss of ATP and cellular membrane damage. As a result, cell integrity is severely disrupted and cellular contents are released that eventually lead to secondary inflammatory responses, with the potential for pathological consequences. A mechanism through which mitochondria mainly contribute to necrosis during pathological conditions is permeability transition, the opening of high‐conductance and long‐lasting mPTP on IMM.

Under physiological conditions, IMM is almost impermeable to all chemicals except a few ions and metabolites, which is crucial for the maintenance of the pH gradient and mitochondrial ∆Ψ. Under pathological conditions, calcium overload and oxidative stress are key factors that induce mPTP opening on IMM. In contrast, it is inhibited by low pH, adenine nucleotides and pharmacological inhibitors such as http://www.guidetopharmacology.org/GRAC/LigandDisplayForward?ligandId=1024 (CsA) (Halestrap *et al*., 2004). Increased mPTP opening causes loss of IMM integrity followed by ∆Ψ dissipation across IMM, which reduces the efficiency of ATP synthesis and induces necrotic cell death. However, upon mPTP opening, only ions, water and other solutes up to 1.5 kDa enter/exit the matrix. Proteins, due to their high MW, are retained in the matrix. Since the concentration of ions (Ca^2+^) in the matrix is less than in the cytoplasm, ions enter the matrix through pores upon mPTP induction. As a result, high colloidal osmotic pressure increases water influx into the matrix, and hence eliciting colloid osmotic pressure. Consequently, the IMM expands and swells in order to resist this pressure. However, the OMM is unable to expand, which ultimately causes mitochondrial membrane rupture and leads to cellular necrosis. In addition, OMM rupture might secondarily cause the release of apoptogens, which activate downstream apoptotic signalling *via* apoptosome assembly and caspase activation (Kwong and Molkentin, 2015). Beyond cell death, mPTPs opening has been proposed to be involved in ROS‐induced ROS release (RIRR), a positive feedback mechanism in which mitochondria increase their own ROS production in response to an elevated level of ROS (Gustafsson and Gottlieb, 2008).

In spite of considerable efforts, the molecular composition and regulation of mPTP has become an area of controversy and remains uncertain. Several studies have identified at least three proteins, cyclophilin D (CypD), VDAC and ANT as critical facilitators for mPTP opening (Murphy *et al*., 2016). CypD is a matrix mitochondrial enzyme, which was first reported to be involved in mPTP function through the investigation of an immunosuppressive drug CsA (Crompton *et al*., 1988). Nowadays, CypD is accepted as an undisputed and indispensable regulator of mPTP, which has been verified using CypD‐deficient mice, which show less sensitivity to Ca^2+^‐ and ROS‐induced mPTP opening (Baines *et al*., 2005; Nakagawa *et al*., 2005). Moreover, pharmacological inhibition of CypD or genetically disrupted expression of *CypD* ameliorated mPTP opening and cell death and protected against I/R injury and muscular dystrophy (Palma *et al*., 2009; Alam *et al*., 2015). However, to‐date the precise mechanism whereby CypD regulates mPTP remains unknown. ANT, a 32 kDa IMM protein, is responsible for importing ADP into mitochondrial matrix in exchange for ATP, while VDAC localizes mainly on OMM to form a pore for low‐specific passage of small hydrophilic molecules (<5 kDa) (Kwong and Molkentin, 2015). Pulldown experiments elucidate that ANT and VDAC are components involved in a pore formation of mPTP *via* interaction with CypD (Crompton *et al*., 1998). Together with other proteins, such as hexokinase in the cytosol, translocator protein on OMM, and creatine kinase in the intermembrane space, a complex of mPTP, is formed (Brenner and Moulin, 2012). However, genetic ablation of any of these genes resulting in increases in Ca^2+^‐ and oxidative stress‐sensitive thresholds of mPTP does not block mPTP opening under various Ca^2+^ levels, suggesting that they are not essential for the function of mPTP (Baines *et al*., 2007; Kwong and Molkentin, 2015).

Furthermore, PiC, an IMM protein which transports organic phosphate (Pi) into the mitochondrial matrix, is a newly suggested potential candidate component of the mPTP due to its ability to trigger mPTP opening and being a CypD binding partner (Kwong and Molkentin, 2015). Although a partial deletion of PiC *via* siRNA does not successfully block mPTP opening, cardiac‐specific deletion of PiC protected hearts against I/R injury by blunting Ca^2+^ overload‐induced mPTP opening, suggesting it regulates mPTP through altering mitochondrial matrix Pi levels (Kwong *et al*., 2014; Kwong and Molkentin, 2015). Recently, mitochondrial http://www.guidetopharmacology.org/GRAC/FamilyDisplayForward?familyId=156#803 has also emerged as a component of mPTP. Giorgio *et al*. (2013) initially showed that the binding of CypD to an oligomycin sensitivity‐conferring protein (OSCP) subunit of the peripheral stalk of ATP synthase led to a modulation of ATP synthase activity. Moreover, a purified dimer of F1F0 ATP synthase reconstitutes to form a Ca^2+^‐activated channel in lipid bilayers, which harbour activity resembling mPTP (Giorgio *et al*., 2013). A further study by Alavian *et al*. (2014) demonstrated that deletion of the c‐subunit of ATP synthase results in the prevention of Ca^2+^‐induced mPTP opening and protects against Ca^2+^‐ and ROS‐induced cell death in cultured neurons. However, two recent findings by the Walker group have challenged these concepts by creating various clonal cells that harboured deletions of genes coding for OSCP and c‐subunits, which showed that the absence of either of these components does not prevent permeability transition in human mitochondria (He *et al*., 2017a; He *et al*., 2017b). Taken together, these findings provide compelling evidence that F1F0 ATP synthase contributes to mPTP function, but the underlying mechanisms require clarification.

Several studies have indicated that the opening of mPTP occurs predominantly during various cardiac diseases. During ischaemia, the anaerobic condition lowers cytosolic pH to acidic values by metabolizing pyruvate into lactic acid, which inhibits mPTP opening (Gustafsson and Gottlieb, 2008). On the other hand, most of the Ca^2+^ pumping channels are blunted due to lack of ATP, and a sudden rise in Ca^2+^ concentration primes for prolonged mPTP opening during I/R injury (Kwong and Molkentin, 2015). During heart failure, impairments in Ca^2+^ homeostasis, ATP depletion and up‐regulated ROS could favour mPTP opening, which might also be a key factor contributing to pump failure. CypD^−/−^ mice displayed improved mortality and reduced infarct size in response to prolonged I/R injury, whereas mice with CypD overexpression had mitochondrial dysfunction and cell death (Baines *et al*., 2005; Lim *et al*., 2010). Despite considerable efforts to address the beneficial effects of inhibiting mPTP, Elrod *et al*. (2010) demonstrated that CypD‐deficient mice exhibit more severe cardiac hypertrophy and fibrosis with reduced function in response to pressure overload. The following experiments showed that these CypD‐deficient hearts had accumulations of Ca^2+^ within the mitochondrial matrix and altered cardiomyocyte metabolism, thereby reducing metabolic flexibility and facilitating the onset of heart failure. These results indicate the importance of mPTP in the regulation of cardiac metabolism through maintaining Ca^2+^ homeostasis, which might be beneficial during the compensation stage of cardiac hypertrophy (Elrod *et al*., 2010). Although mPTP opening contributes significantly to the progression of heart failure, further studies are required to fully explore the molecular structure and regulation of mPTP in order to develop novel therapeutic strategies for treating heart disease.

### Necroptosis

Recently, the conventional dogma of necrosis in cardiac disease is changing with a concept of programmed and regulated necrosis, termed necroptosis. Under pathological conditions, the initiation of necroptosis is highly associated with an inflammatory response through activation of the http://www.guidetopharmacology.org/GRAC/LigandDisplayForward?ligandId=5074 signalling pathway. This is initiated by the binding of http://www.guidetopharmacology.org/GRAC/ObjectDisplayForward?objectId=2189) to http://www.guidetopharmacology.org/GRAC/ObjectDisplayForward?objectId=2191, causing oligomerization and autophosphorylation of RIP3 to form necrosomes. The activated necrosomes in turn mediate phosphorylation of http://www.guidetopharmacology.org/GRAC/ObjectDisplayForward?objectId=2106) and its translocation to the plasma membrane to form pores, eventually disrupting the integrity of the cell membrane and promoting necrosis (Galluzzi *et al*., 2017).

In addition, Wang *et al*., 2012 showed that mitochondrial fragmentation is a mediator of necroptosis. Upon TNF‐α induction, the mitochondria phosphatase PGAM5, a part of RIPK1‐RIPK3‐MLKL complexes, becomes activated leading to the recruitment and dephosphorylation of Drp1. Consequently, excessive GTPase activity of Drp1 results in an increased mitochondrial fragmentation, which is required for early events of necroptosis. However, further investigation indicated that activation of PGAM5 is cardioprotective against necroptosis in acute stress by independently activating PINK1‐mediated mitophagy. Cardiomyocytes from *Pgam5* ^*−/−*^ mice showed an accumulation of damaged mitochondria, having greater levels of necroptosis and were more susceptible to IR injury (Lu *et al*., 2016). These data reveal different facets of the role of mitochondria in necroptosis.

Numerous studies have indicated the role of necroptosis in heart diseases, including myocardial infarction and heart failure. In patients with failing hearts, increased levels of TNF‐α lead to activation of RIPK1‐RIPK3‐mediated necroptosis. Moreover, several necroptotic markers including RIPK1, RIPK3 and phosphor‐MLKL become significantly increased in human end‐stage heart failure (He *et al*., 2009). Several animal models have also confirmed the existence of necroptosis and its crucial role during the development of heart failure, and necroptosis inhibition seems to be beneficial and alleviate cardiac dysfunction. For example, pharmacological inhibition of RIPK1 activity by http://www.guidetopharmacology.org/GRAC/LigandDisplayForward?ligandId=9750 prevented necroptosis and ameliorated I/R injury in mice and rats (Anbin *et al*., 2014). Inhibition of RIPK1 and RIPK3 also prevents cardiomyocyte necroptosis‐mediated cell death in response to hypertrophy‐induced lipotoxicity stress (Zhao *et al*., 2016). Thus, understanding the pathological role of necroptosis sheds light on a new strategy to treat heart disease; however, the molecular mechanisms underlying necroptosis in the pathogenesis of heart diseases are needed to be fully delineated.

### Therapeutic approaches targeting mitochondria

**Figure 3:**
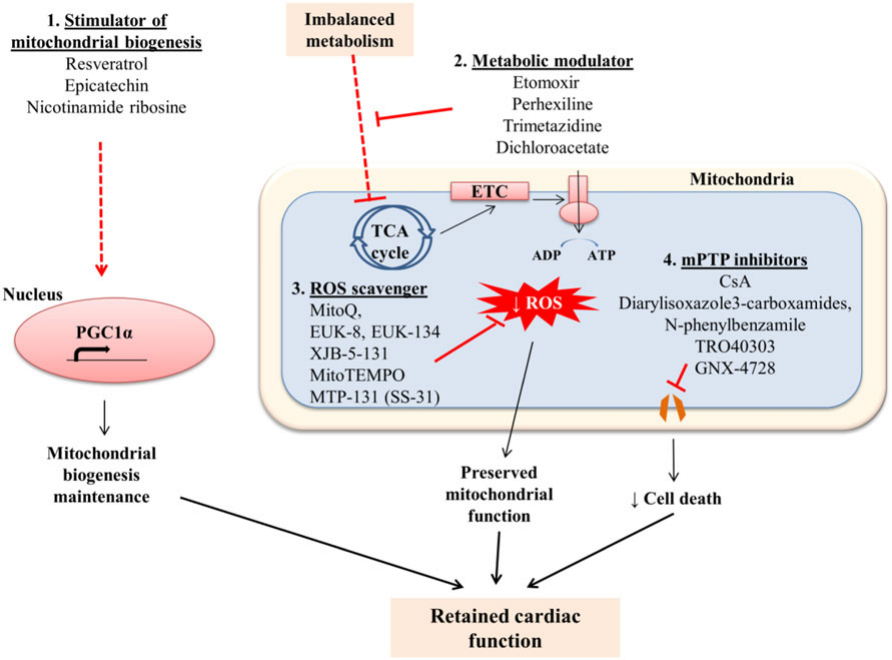
Preserving mitochondrial biogenesis and function as therapeutic approaches in cardiac diseases. The potential therapeutic strategies aim to facilitate mitochondrial biogenesis and maintain mitochondrial function to prevent cardiac dysfunction, including (i) enhancing mitochondrial biogenesis; (ii) retaining mitochondrial metabolism by limiting fatty acid import and oxidation while improving glucose utilization; (iii) reducing ROS production by ROS scavengers; and (iv) inhibiting mPTP by the use of mPTP inhibitors.

Given the important roles of mitochondria in cardiomyocyte homeostasis and cardiac function, mitochondrial‐based therapies have been extensively studied. These therapeutic approaches include stimulators of mitochondrial biogenesis, metabolic modulators, ROS scavengers, mPTP inhibitors and gene therapy by targeting the deregulated mitochondrial‐related genes (Figure 3). Each strategy will be further discussed as follows.

### Stimulators of mitochondrial biogenesis

The first therapeutic approach focuses on improving mitochondrial biogenesis and thus mitochondrial function, which results in the preservation of cardiac function. Polyphenol compounds such as http://www.guidetopharmacology.org/GRAC/LigandDisplayForward?ligandId=8741 and epicatechin activate SIRT1/3 to promote AMPK and http://www.guidetopharmacology.org/GRAC/ObjectDisplayForward?objectId=1249 signalling, which are responsible for regulating mitochondrial biogenesis through the activation of mitochondrial transcription factors, PGC1α and NRFs (Brown *et al*., 2017). The beneficial effects of resveratrol have also been proven in recent studies on coronary artery disease, atherosclerosis, hypertension and myocardial infarction (Berman *et al*., 2017). In a 3‐month randomized, double‐blind, placebo‐controlled clinical trial, resveratrol, 10 mg·day^−1^, was shown to improve systolic function and reduce LDL‐cholesterol and platelet aggregation in patients with myocardial infarction (Bonnefont‐Rousselot, 2016). However, its short half‐life of 8–14 min within the body is the main challenge to deliver an effective dose in humans. Therefore, the development of other derivatives with longer half‐lives would offer better treatment for patients with myocardial infarction. Meanwhile, catechins are the key regulators of systemic blood pressure, atherosclerosis, platelet activation and thrombosis formation (Mangels and Mohler, 2017). The 25 year Zutphen Elderly Study has indicated that dietary epicatechin, mainly found in cocoa, black and green teas, lowers mortality in elderly Dutch men with prevalent cardiovascular disease (Dower *et al*., 2016). Two large clinical trials, namely, the COCOA‐BP trial and the FLAVASCULAR study, are currently underway to assess the effect of catechin‐rich food on regulating systemic BP (NCT02764203; NCT02013856).

In addition to these compounds, another natural compound nicotinamide riboside (NR) can be utilized as a diet supplement to enhance NAD^+^ availability, leading to SIRT1 activation. The up‐regulated deacetylase activity of SIRT1 in turn improves mitochondrial function *via* PGC1α cascade (Viscomi *et al*., 2015). A NR‐supplemented diet has been shown to improve NAD^+^ levels, thus attenuating the progression to heart failure in both mouse models of dilated cardiomyopathy and of cardiac hypertrophy (Diguet *et al*., 2017). A recent clinical trial has demonstrated that 6 weeks of NR supplementation induces a significant increase in blood NAD^+^ concentration and was well‐tolerated in healthy middle‐aged people (Martens *et al*., 2018). Another double‐blind clinical trial is currently underway to investigate the safety and tolerability of NR in patients with clinically stable systolic heart failure (NCT03423342).

### Metabolic modulators

As mentioned earlier, myocardial energy metabolism undergoes dramatic alterations in glycolysis, FAO capacity and ATP production under pathological stresses. To explore potential therapeutic strategies by reserving cardiac energy metabolism, inhibition of fatty acid uptake, inhibition of FAO and stimulation of glucose oxidation are currently under investigation.

The first strategy aims mainly to limit the adverse effects of accumulated fatty acids in the heart and thus cardiac lipotoxicity through inhibiting the key fatty acid importer carnitine palmitoyltransferase‐1 (CPT1). CPT1 is mainly responsible for mitochondrial import of fatty acids, thus it determines the balance between fatty acid and glucose oxidation within the cardiomyocyte mitochondrial matrix. CPT1 inhibition by http://www.guidetopharmacology.org/GRAC/LigandDisplayForward?ligandId=9089 and perhexiline, which have been shown to improve cardiac output and the exercise capacity of patients with heart failure, but their use remains limited due to side effects such as peripheral neuropathy and hepatoxicity (Lee *et al*., 2005; Holubarsch *et al*., 2007).

The second strategy is achieved using the pharmacological compound trimetazidine, an inhibitor of long‐chain 3‐ketoacyl CoA thiolase, which is involved in the last step of FAO in mitochondria (Kantor *et al*., 2000). In ischaemic hearts, trimetazidine can restore the balance between glycolysis and glucose oxidation to preserve energy production with limited oxygen supply. Furthermore, this compound also exerts other beneficial effects, such as the induction of pyruvate dehydrogenase in the TCA cycle, inhibition of hypoxia/reperfusion injury and inhibition of cardiac fibrosis (McCarthy *et al*., 2016). Trimetazidine is currently used for treating angina in more than 100 countries and has been shown to improve cardiac function and the quality of life in patients with both non‐ischaemic and ischaemic heart failure (Gao *et al*., 2011). A combination of trimetazidine and β‐blockers for angina treatment has also been tested in three different clinical trials, which have shown that 12 week trimetazidine administration in conjunction with β‐blockers could increase time to onset of angina pain and exercise capacity compared to the group treated with only β‐blockers (Vitale *et al*., 2013). Moreover, a small clinical trial involving 64 patients with stable angina revealed that a combination of trimetazidine and calcium channel blockers reduces the number of angina attacks without adverse haemodynamic effects (Manchanda and Krishnaswami, 1997). In addition to angina treatment, trimetazidine also offers clinical benefits in patients with diabetes mellitus, coronary artery disease and heart failure, although these effects require confirmation in larger clinical trials (Dézsi, 2016).

The third strategy focuses on enhancing myocardial glucose oxidation to meet increased cardiac glycolysis in the failing heart through use of the pharmacological agent http://www.guidetopharmacology.org/GRAC/LigandDisplayForward?ligandId=4518, a PDK inhibitor (Bersin and Stacpoole, 1997). Inhibition of PDK thus reduces its inhibitory effect on pyruvate dehydrogenase, a key enzyme involved in pyruvate oxidation, thus allowing increased glucose oxidation in ischaemic cardiomyocytes. Using a rat model, dichloroacetate pretreatment retains cardiac function and glucose oxidation in response to ventricular fibrillation‐induced ischaemic. However, some studies challenged that, *via* the Randle cycle, increased glucose metabolism could lead to reduced fatty acid metabolism; thus, the accumulation of lipid metabolites such as triglycerides, diglycerides and ceramide might cause cardiac lipotoxicity (Fillmore and Lopaschuk, 2013). Therefore, this strategy requires further investigation and validation.

### ROS scavengers

Reducing ROS production is a theoretical strategy to treat cardiac pathological remodelling and heart failure. However, the clinical implications need to be further inspected. In a large clinical trial involving 765 middle‐aged men, often‐used antioxidants such as vitamin E or C failed to reduce the risk of cardiovascular events, suggesting that these compounds might not get to the site of ROS generation (Sesso *et al*., 2008). So far, MitoQ, a ROS scavenger linked to triphenylphosphonium, is the best characterized mitochondria‐targeted antioxidant. By accumulating in the negatively charged mitochondrial matrix, lipophilic triphenylphosphonium cations of MitoQ effectively recruit CoQ10 to the mitochondrial matrix in order to improve ETC efficiency and reduce ROS production (Bayeva *et al*., 2013a). However, depolarization of mitochondrial matrix induced by the accumulation of cationic triphenylphosphonium could potentially interfere with mitochondrial function in the heart (Bayeva *et al*., 2013a). In various preclinical studies, MitoQ appears to be protective in animal models with hypertension, I/R injury and pressure overload stress (Ribeiro *et al*., 2018). In a small clinical trial involving 55 middle‐aged and older adults, MitoQ treatment reduced aortic stiffness, plasma oxidized LDL and improved vascular endothelial function (Rossman *et al*., 2018). However, the uptake of MitoQ is dependent on the ∆Ψ, which is dramatically disrupted in human failing hearts (Bayeva *et al*., 2013a). This remains the key challenge for the therapeutic application of MitoQ for heart failure.

In addition to MitoQ, other ROS‐scavenging compounds have been rigorously tested in different heart disease models. For example, EUK‐8, a SOD and catalase mimetic, has been shown to protect mouse hearts against pressure overload‐induced heart failure and dilated cardiomyopathy (Empel *et al*., 2006; Kawakami *et al*., 2009). EUK‐134, a more lipophilic derivative of EUK‐8, blunts hypertrophic responses in H9C2 cardiac cell lines (Purushothaman and Nair, 2016). EUK‐134 also prevents diaphragm muscle weakness in the rat model of monocrotalin‐induced pulmonary hypertension (Himori *et al*., 2017). Also, XJB‐5‐131, an antioxidant which scavenges electrons that have leaked from the ETC, blocks the oxidative stress response and improves mitochondrial respiratory function in aged rat skeletal muscle and aged I/R stressed hearts (Escobales *et al*., 2014; Javadov *et al*., 2015). Moreover, MitoTEMPO, a mitochondrial‐targeted ROS scavenger, ameliorates hypertension and enhances endothelial NO production in mice infused with angiotensin II (Dikalova *et al*., 2010). However, since studies on the above compounds are mostly based on transgenic animal models, further clinical investigation into their safety and efficacy is required.

The IMM phospholipid, cardiolipin, is important for regulating ETC activities, mitochondrial membrane transporters and ROS generation. To overcome non‐selective delivery of other ROS scavengers, small mitochondrial‐targeted peptides have recently been developed to enter the mitochondrial matrix independently of membrane potential. MTP‐131 (also known as SS‐31 or Bendavia), a novel pharmacological compound targeting cardiolipin, protects the heart against I/R injury in rabbit and sheep models (Kloner *et al*., 2012); it also prevents pressure overload‐induced heart failure through suppression of cardiac proteomic remodelling and cardiomyocyte necrosis (Dai *et al*., 2013; Nickel *et al*., 2015). Although MTP‐131 appears to be well‐tolerated in phase I and II clinical trials, the phase 2a EMBRACE STEMI study has indicated that treatment with MTP‐131 at a dose of 0.05 mg·kg^−1^·h^−1^ cannot reduce myocardial infarct size in STEMI patients undergoing percutaneous coronary intervention (Gibson *et al*., 2016). Meanwhile, in a double‐blind, placebo‐controlled, ascending‐dose trial involving patients suffering from heart failure with reduced ejection fraction, MTP‐131 treatment at a dose of 0.25 mg·kg^−1^·h^−1^ significant decreases left ventricular end‐diastolic volume and end‐systolic volume (Daubert *et al*., 2017). Therefore, further studies are essential to assess its effect for the treatment of specific heart diseases.

### mPTP inhibitors

Inhibition of mPTP presents a promising therapeutic strategy to prevent cardiomyocyte death and mitochondrial dysfunction. CsA, a CypD inhibitor, has been previously shown to protect the Langendorff‐perfused hearts from re‐oxygenation and perfusion injury (Griffiths and Halestrap, 1993). CsA administration also enhances mitochondrial respiratory function and ∆Ψ in isolated cardiomyocytes from dogs with heart failure (Sharov *et al*., 2007). In a phase II clinical trial, administration of CsA immediately before percutaneous coronary intervention reduces infarct size in patients with acute anterior ST‐segment elevation myocardial infarction (STEMI) (Piot *et al*., 2008). However, in a following phase III trial involving 970 patients with STEMI, intravenous injection of CsA failed to improve clinical outcome or prevent adverse ventricular remodelling at 1 year compared to the group with placebo treatment (Cung *et al*., 2015). The failure of CsA treatment is likely due to its inhibition of CypD, which might disturb CypD‐dependent cardiac mitochondrial Ca^2+^ homeostasis and cellular energy metabolism (Javadov *et al*., 2017). Of note, this compound also displays severe side effects such as immunosuppression and adverse effects on renal pathology (Brown *et al*., 2017), plausibly making CsA application an inappropriate treatment for heart diseases.

With the interest in suppressing mPTP‐induced cell death in ischaemic hearts, new mPTP blockers have recently been developed to selectively inhibit mPTP opening with few adverse effects on CypD. For example, diarylisoxazole3‐carboxamides, as potent inhibitors of mPTP, increase Ca^2+^ retention in mitochondria and prevent mitochondrial swelling in zebrafish muscle (Roy *et al*., 2015). *N*‐phenylbenzamide, a compound also identified as a mPTP‐specific blocker through high‐throughput screening, inhibits mitochondrial swelling in mouse liver mitochondria and HeLa cells (Roy *et al*., 2016). Furthermore, cinnamic anilides have been developed as a novel class of mPTP inhibitors, which exert protective effects in the rabbit acute myocardial infarction model (Fancelli *et al*., 2014). Chronic treatment with GNX‐4728, a cinnamic anilide compound, can also be a potential treatment for amytrophic lateral sclerosis in mice (Martin *et al*., 2014). However, in a recent MITOCARE clinical trial, another mPTP inhibitor TRO40303 failed to show efficacy in limiting cardiac injury in patients with STEMI (Atar *et al*., 2015). Taken together, further studies on these newly discovered mPTP blockers are required to comprehensively explore their therapeutic potential for treating heart diseases.

### Gene therapy

Mitochondrial dysfunction in cardiac disease and metabolic syndromes are highly associated with a down‐regulation of key proteins responsible for mitochondrial biogenesis and function such as PGC1α and NRFs (discussed in ‘Mitochondrial transcriptional network' section). Gene therapy is a novel approach to restore the expression of critical proteins responsible for mitochondrial function *via* adeno‐associated virus serotypes (AAVs) delivery system. AAVs are small, non‐pathogenic viruses with a single‐strand DNA genome. One major advantage of using recombinant AAVs for research is that the transduced gene does not integrate into the host's genome and the transgene expression level is controllable by adjusting the amount of virus injected (Viscomi *et al*., 2015). Among AAV serotypes, AAV9 can efficiently deliver a transgene under the control of a cardiac‐specific promoter into cardiomyocytes. Liu *et al*. (2017) reported that ERK5 is a requisite for sustaining PGC1α expression and the restoration of ERK5 expression by the AAV9 system ameliorates mitochondrial function and prevents high‐fat‐diet‐induced cardiomyopathy.

## Conclusion

Mitochondrial biogenesis in the myocardium is closely related to cardiac physiological function. However, myocardial mitochondrial function is damaged under various stresses, leading to pathological cardiac remodelling and heart failure. Mitochondrial dysfunction contributes to the development of cardiomyopathies through energy deprivation, the accumulation of ROS and cell death. Designing therapeutic strategies targeted to maintain mitochondrial function has been challenging due to the different responses observed that depend on the aetiology of the disease. Nonetheless, the continuous advancement in our knowledge of the molecular basis underlying mitochondrial biogenesis in physiological and pathological conditions is being pursued in order to discover novel therapeutic targets for heart diseases.

### Nomenclature of targets and ligands

Key protein targets and ligands in this article are hyperlinked to corresponding entries in http://www.guidetopharmacology.org, the common portal for data from the IUPHAR/BPS Guide to PHARMACOLOGY (Harding *et al*., 2018), and are permanently archived in the Concise Guide to PHARMACOLOGY 2017/18 (Alexander *et al*., 2017a, 2017b, 2017c).

## Conflict of interest

The authors declare no conflicts of interest.
